# Joint angle-specific neuromuscular time course of recovery after isometric resistance exercise at shorter and longer muscle lengths

**DOI:** 10.1152/japplphysiol.00820.2023

**Published:** 2024-03-07

**Authors:** Gerard McMahon, Gladys Onambele-Pearson

**Affiliations:** ^1^Sport and Exercise Sciences Research Institute, School of Sport, Ulster University, Belfast, United Kingdom; ^2^Research Centre for MusculoSkeletal Sciences & Sport Medicine, Department of Sport and Exercise Sciences, Manchester Metropolitan University, Manchester, United Kingdom

**Keywords:** activity, creatine kinase, moment arm, quadriceps, strength

## Abstract

Resistance training at longer muscle lengths induces greater muscle hypertrophy and different neuromuscular functional adaptations than training at shorter muscle lengths. However, the acute time course of recovery of neuromuscular characteristics after resistance exercise at shorter and longer muscle lengths in the quadriceps has never been described. Eight healthy young participants (4 M, 4 F) were randomly assigned to perform four sets of eight maximal isometric contractions at shorter (SL; 50° knee flexion) or longer (LL; 90° knee flexion) muscle lengths in a crossover fashion. During exercise, peak torque (PT), muscle activity [electromyogram (EMG)], and internal muscle forces were assessed. PT and EMG at shorter (PT50, EMG50) and longer (PT90, EMG90) muscle lengths, creatine kinase (CK), and muscle soreness were measured at baseline, immediately after exercise (Post), after 24 h (24 h), and after 48 h (48 h). During exercise, EMG (*P* = 0.002) and internal muscle forces (*P* = 0.017) were greater in LL than in SL. During recovery, there was a main effect of exercise angle, with PT50 (*P* = 0.002), PT90 (*P* = 0.016), and EMG50 (*P* = 0.002) all significantly reduced to a greater degree in LL compared with SL. CK and muscle soreness increased after resistance exercise, but there were no differences between SL and LL. The present results suggest that if the preceding isometric resistance exercise is performed at longer muscle lengths, function and muscle activity at shorter and longer muscle lengths are inhibited to a larger degree in the subsequent recovery period. This information can be used by practitioners to manipulate exercise prescription.

**NEW & NOTEWORTHY** Despite the established long-term benefits of training at longer muscle lengths for muscle size and strength, acutely performing resistance exercise at longer muscle lengths may require a longer time course of neuromuscular recovery compared with performing resistance exercises at shorter muscle lengths. Furthermore, there appear to be different joint angle-specific recovery profiles, depending on the muscle length of the preceding exercise.

## Graphical abstract

**Figure:**
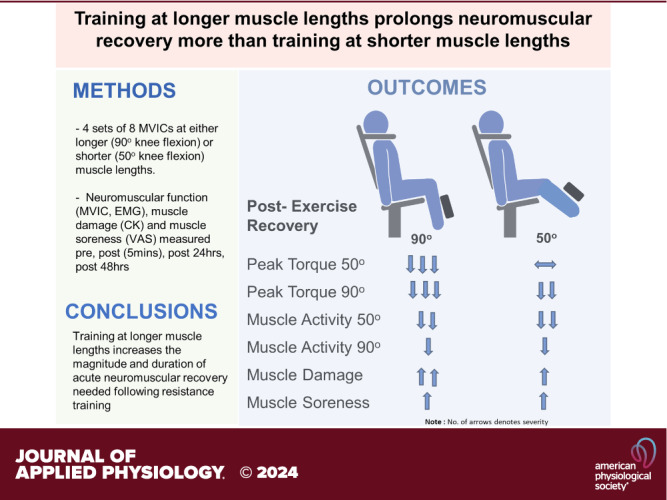

## INTRODUCTION

Performing volume-matched dynamic and isometric resistance training at longer muscle lengths has been shown to improve muscle cross-sectional area, muscle architecture, and general or joint angle-specific strength and tendon mechanical properties to a greater extent than training at a shorter muscle length (1–5). However, voluntary activation (6–8), muscle activity (9), and muscle oxygen consumption (6, 10) are greater, and time to fatigue is shorter (11–15), when training isometrically at longer muscle lengths compared with shorter muscle lengths in the knee extensors. The increased metabolic and neuromuscular demands of producing isometric force at longer muscle lengths may be largely reflective of the vast differences in internal muscle mechanics between joint angles. At the knee, the patella tendon moment arm at 90° knee flexion is <50% of the length of the moment arm at 50° knee flexion (16). Mechanically, this results in a two- to threefold higher internal muscle force production at 90° knee flexion to generate the same external torque at 50° (5). As a result, these greater neuromechanical and metabolic demands of exercising at longer muscle lengths, although beneficial over more extended periods of training, may prolong postexercise recovery.

Persistent force depression is a common feature of recovery periods after resistance training. Various mechanisms can lead to inhibition of contractile machinery performance, such as ultrastructural damage, residual metabolic products from contractions, and ionic changes from repeated activations and opening of stretch-activated ion channels (17–19). Jones et al. (20) showed that after a maximal voluntary isometric contraction (MVIC) every 15 s for 20 min at either shorter or longer elbow flexor muscle lengths, postexercise peak force was significantly lower after long-muscle length exercise. There was significantly greater low-frequency fatigue, which is the relative loss of force at low frequencies of muscle stimulation (21), and pain of the elbow flexors reported 72 and 96 h after exercise at longer versus shorter muscle lengths. Philippou and colleagues (22, 23) found that performing maximal isometric contractions at a longer muscle length (155° elbow angle) resulted in a sustained decrement of maximal isometric force at shorter muscle lengths (50–90° elbow angle) for up to 4 days after exercise, whereas maximal isometric force at longer muscle lengths was either unaffected or had recovered to baseline levels within the 4-day recovery period. However, there was no comparative shorter-muscle length condition in this study, with the effects of longer-muscle length exercise investigated in isolation. Allen et al. (24) had participants perform maximal isometric resistance exercise at shorter (90° elbow angle) and longer (155° elbow angle) muscle lengths in the elbow flexors. Their results showed that exercise in both muscle length conditions resulted in significant reductions in MVIC immediately after and 2 h, 4 h, and 24 h after exercise at their respective joint angles. The authors noted a significant effect of exercise condition at each of the recovery time points, with the change in maximal torque relative to baseline being significantly higher in the longer-muscle length condition compared with shorter muscle length. However, the comparisons of torque measured during the recovery time frame were matched to the preceding exercise condition only and then compared (i.e., after exercise at 90° torque was only assessed at 90° and after exercise at 155° torque was assessed at 155°). The authors did not assess how either shorter- or longer-muscle length exercise affected function at any joint angles other than the training angle. Therefore, neither the studies of Philippou et al. (22, 23) nor those of Allen et al. (24) allow for the identification of the full impact of exercising at shorter and longer muscle lengths on the resulting recovery of muscle function at both shorter and longer muscle lengths. This is a central consideration, because after resistance training sarcomere dynamics can be acutely affected during recovery, where their operating lengths may be switched to longer lengths, altering how the muscle functions depending on the joint angle used during training (25). In a practical sense, this information is important, as individuals performing multiple resistance training sessions per week may wish to alter joint angles or range of motion (ROM) to avoid having to cease training altogether to facilitate recovery.

Therefore, the primary aim of the present study was to describe the time course of recovery after resistance training protocols at shorter versus longer muscle lengths and the effects on muscle strength at both shorter and longer muscle lengths, activity, damage, and soreness. A secondary aim of the study was to systematically outline the acute neuromuscular impact of training at different muscle lengths to provide evidence of distinctly different exercising condition effects, rather than assuming them from previous evidence. This then will provide greater objectivity and reduce speculation when interpreting the primary objective data. Previous studies have shown that joint angle-specific strength may be underpinned by neurological changes, with different muscle activity states at differing muscle lengths (1, 2), and as noted above ionic changes and metabolic products from repeated activations may inhibit force production. Therefore, the acute neuromuscular impact of training will include electromyographic (EMG) measurements to quantify muscle activity during the exercise protocol. Furthermore, training at different muscle lengths induces important muscle architectural changes [e.g., fascicle length (26)]. As proof of principle that the exercise protocol at different muscle lengths will elicit distinct architectural configurations during the resistance exercise, muscle architectural parameters were assessed. Finally, measures of external torque and calculation of internal muscle forces were also performed. This is important, as the patella tendon moment arm length differences between knee joint angles at longer and shorter muscle lengths create higher mechanical demands at longer muscle lengths (16), so if the recovery from such training is to be assessed, then it is important to quantify the mechanical demands imposed on the muscle during the exercise training itself. It was hypothesized that performing resistance exercise at longer muscle lengths will result in a greater impairment of muscle force, in terms of magnitude or duration of decrement over the recovery time course, at both muscle lengths compared with performing exercise at shorter muscle lengths. It was also hypothesized that the decrement in muscle force will be mirrored in the electromyographic, creatine kinase, and self-reported soreness measures.

## METHODS

### Participants

An a priori power calculation was performed in G*Power software (version 3.1.9.7) using the maximal voluntary contraction (MVC) mean and standard deviations from Allen et al. (24), as MVC is the primary outcome measure of the present study. Also, the participants of Allen et al. (24) trained at a longer and a shorter muscle length with the same design and number of time points as in the present study. The preexercise, immediately postexercise, post-2 h, and post-24 h MVC data from Allen et al. (24) were 58.4 ± 6.5, 49.5 ± 5.7, 53.2 ± 5.9, and 54.9 ± 6.6 Nm (mean ± SD), respectively, for shorter muscle lengths and 61.3 ± 6.7, 41.1 ± 4.9, 48.6 ± 5.2, and 48.7 ± 5.3 Nm, respectively, for longer muscle lengths. The software was set to repeated-measures ANOVA with within-between interaction, to calculate a sample size that would show a medium effect size with an alpha level of 0.05 and beta set at 0.8 across four repeated-measures time points. The results showed that a sample size of eight participants would yield a power of 0.86. A total of eight participants (4 females) volunteered to take part in the study (see Table 1 for participant characteristics). Participants were recruited via convenience sampling from the local university campus and gyms with posters, e-mails, and word of mouth. Participants are described as recreationally active, taking part in regular physical activity two to three times per week (e.g., playing university sports) but not taking part in any formal lower body resistance programs currently or 6 mo before the beginning of the study. To be eligible for the study, individuals needed to be between 18 and 39 yr of age, not have any musculoskeletal or neurological disorders, be free from injury, and not supplementing with any ergogenic aids either 3 mo before or during the study. After a prescreening physical activity questionnaire to ensure eligibility, participants were provided with an information sheet, outlining the full experimental procedure. Subjects were informed of the benefits and risks of the investigation before signing an institutionally approved informed consent document to participate in the study. The study was conducted in accordance with the Declaration of Helsinki, and the protocol was approved by the Ulster University School of Sport Ethics Committee.

**Table 1.** Participant characteristics

	Male (*n* = 4)	Female (*n* = 4)	Whole Sample (*n* = 8)
Weight, kg	91.2 ± 7.1	66.5 ± 15.0	78.8 ± 17.1
Height, cm	180.5 ± 3.9	161.3 ± 4.6	170.9 ± 11.0
Age, yr	23.0 ± 1.2	23.3 ± 2.6	23.1 ± 1.9
Tibial length, cm	39.8 ± 0.4	37.3 ± 0.5	38.6 ± 1.4

Values are means ± SD.

### Study Design

**Figure 1.**
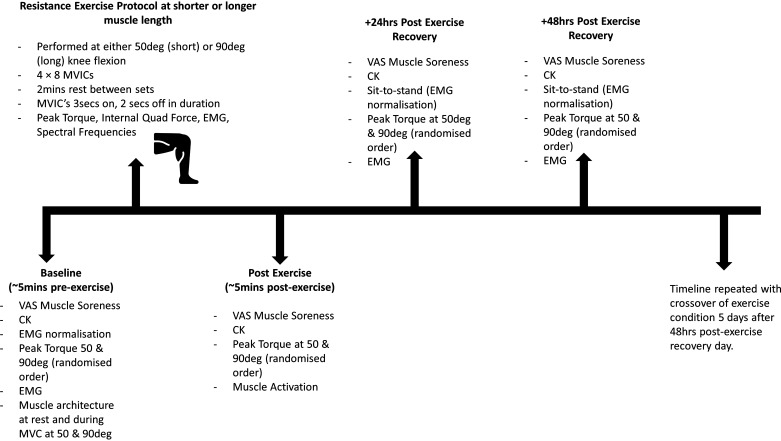
Schematic of the study design. CK, creatine kinase; EMG, electromyogram; MVC, maximal voluntary contraction; MVIC, maximal voluntary isometric contraction; VAS, visual analog scale.

This study used a within-subjects, randomized, counterbalanced study design, which included seven experimental visits to the laboratory. The first laboratory visit included familiarizing participants with the experimental exercise protocols. Each participant completed three maximal isometric voluntary contractions (MVICs) of unilateral knee extensions at both 50° and 90° joint angle (0° = full extension of knee) separated by 1-min rest after a brief warm-up (2 sets of 5 repetitions at 50% and 80% perceived MVIC, respectively). After a minimum of 3 days of rest, participants were randomly allocated to an experimental resistance exercise condition, whereby four sets of eight unilateral knee extension MVICs separated by 2-min interset recovery were completed at either a shorter muscle length (50° knee flexion; SL) or a longer muscle length (90° of knee flexion; LL). The last day of the first experimental condition and the first day of the second experimental condition were separated by 5 days. Neuromuscular characteristics (peak torque at both 50° and 90° knee flexion, muscle activity), muscle damage [creatine kinase (CK)], and muscle soreness perception were assessed at baseline (5 min immediately before resistance exercise session) and then at 5 min, 24 h, and 48 h after resistance exercise, respectively (see Fig. 1 for details).

### Knee Extensor Maximal Voluntary Isometric Contraction

Maximal voluntary isometric contractions (MVICs) were performed at baseline (5 min before resistance exercise protocols), after exercise (5 min after cessation of last repetition of resistance exercise protocols), and then at 24 and 48 h after cessation of resistance exercise. At each time point, MVIC was assessed at both 50° and 90° knee flexion in a randomized order. Before this assessment, measures were taken to minimize inaccuracies during the dynamometer (Kin Com, Chattanooga, TN) assessments, thereby counteracting any potential effect of soft tissue compliance, dynamometer alignment, as well as gravitational forces. With these precautions in place, maximal isometric knee extension torque was measured at the selected joint angle on the right leg of all participants, corresponding to the angle of peak torque. After a series of warm-up trials consisting of 10 submaximal isometric contractions, progressed at a self-perceived 50–90% maximal effort, participants were instructed to rapidly exert maximal knee extensor isometric force against the dynamometer lever arm, with a sampling frequency of 250 Hz. Isometric contractions were held for ∼2–3 s at the plateau, with a 60-s rest period between the two contractions at each joint angle and 2-min rest between joint angles. Instantaneous peak torque (PT) was displayed on the dynamometer display screen, providing visual feedback to participants. The highest value of the two contractions was used taken for peak torque, with a coefficient of variation of <5% for all participants between the first two trials meaning that no further repeat trials were required. PT50 denotes peak torque produced at 50° knee flexion, and PT90 denotes peak torque produced at 90° knee flexion.

### Muscle Architecture

Vastus lateralis (VL) muscle architectural measurements were taken at rest and during MVIC with each participant seated in an upright position on an isokinetic dynamometer. After calibration, each participant was positioned with a hip angle of 80° (straight back = 90°) and knee at 90° knee flexion (straight leg = 0°). All measurements were determined with ultrasonography (ArtUs EXT-1H; Telemed, Vilnius, Lithuania) with a 60-mm probe length and screen recorded at 40 frames per second with screen capture software (OBS Studio, Lain; www.obsproject.com) on a PC laptop (Dell Latitude 5420; Dell Inc., Texas). The measurement site was 50% of femur length. Femur length was defined as the line passing from the greater trochanter to the central palpable point of the space between the femur and tibia heads when the knee was flexed at 90°. Vastus lateralis fascicle pennation angle (PA) was measured as the angle of fascicle insertion into the deep aponeurosis. Images were obtained perpendicular to the dermal surface of the VL and oriented along the midsagittal plane of the muscle. The transducer was then aligned in the fascicle plane to capture an optimal number of clearly demarked fascicles. Images were taken at 50% of the total femur length and 50% of muscle width at each point (where 50% muscle width is defined as the midpoint between the fascia separating the VL and rectus femoris and fascia separating the VL and biceps femoris muscles). Fascicle length (Lf) was defined as the length of the fascicular path between the deep aponeurosis and superficial aponeurosis of the VL. When the majority of the length of a fascicle extended off the acquired image, the missing portion was estimated by linear extrapolation. This was achieved by measuring the linear distance from the identifiable end of a fascicle to the intersection of a line drawn from the fascicle and a line drawn from the superficial aponeurosis (see Supplemental Fig. S1, available at https://doi.org/10.6084/m9.figshare.25270375). This method has been shown to produce reliable results previously (27). In the present study, coefficients of variation for muscle thickness, fascicle length, and pennation angle were 2.0%, 2.9%, and 2.6%, respectively, for within-day reliability and 2.4%, 4.1%, and 3.3%, respectively, for between-day reliability. All images were analyzed and measured with ImageJ v.1.43c (National Institutes of Health, Bethesda, MD). After calibration in ImageJ to coincide with the scale of the ultrasound image, a line from the top to the bottom of the superficial to deep aponeurosis visualized was drawn at three regular intervals on the ultrasound image. The average lengths of these three lines were taken to estimate the average thickness of muscle thickness in centimeters. Care was taken not to deform or compress the muscle, with minimal pressure applied to the dermal surface with the ultrasound probe. Images were taken at rest and during the MVICs at baseline before exercise at both muscle lengths. During MVICs the images analyzed synchronized with the time of peak torque, with the average architectural measurements of three frames around time of peak torque used to define muscle architecture.

### Electromyography

Electromyograms were recorded from the vastus lateralis muscle of the quadriceps at 50% of femur length. Each electrode’s bipolar arrangement was positioned parallel to the muscle’s fascicle orientation. To ensure that electrodes were located in the same place for each condition, the location described above was marked with a 2-cm crosshair with an anatomical pen, which remained clearly visible for the duration of the study. Regardless, each crosshair was checked for accuracy during each visit as a precaution. Before electrodes were attached, all locations were prepared by shaving and abrading with a commercially available dermal scrub (St. Ives; UniLever, Wirral, UK) and cleansed with an alcohol swab (70% ethanol) to reduce skin impedance. Each electrode was secured with a combination of the system manufacturer’s own specific electrode double-sided adhesive tape and further fixation with additional adhesive tape.

Surface EMG signals were acquired at a sample rate of 2,000 Hz with a Delsys Wireless Trigno system (Delsys Inc., Boston, MA) connected to a digital data acquisition unit (PowerLab 16/35; ADInstruments, Oxford, UK). One bipolar Trigno sensor was used per muscle site, with each sensor containing Ag/AgCl electrodes with a 10-mm interelectrode distance, with a dual on-board stabilizing reference. The system filtered the EMG data with a fourth-order zero-lag Butterworth filter from 20 to 450 Hz. Raw EMGs were then processed as root mean square (RMS) over the entire duration of each muscle contraction with LabChart v8 software (ADInstruments). Peak (200 ms in total, 100 ms either side of the single instantaneous peak data point) and mean (3 s) RMS EMG were obtained from each contraction. As it is inappropriate to compare processed EMGs across multiple days/conditions, the mean RMS EMG from each contraction was normalized by dividing it by the peak RMS EMG elicited during the preexercise MVIC at the allocated resistance exercise experimental joint angle recorded on the same experimental day (i.e., if the experimental angle was 50°, all exercise contractions were normalized to the preexercise MVIC at 50°). This was also the method employed to identify peak EMG activity for each contraction during the resistance exercise. A spectral analysis was also simultaneously performed, with a fast Fourier transformation applied to the raw EMG signal to provide the power spectral density, from which the mean and median frequency of each contraction during the resistance exercise sessions were assessed. As MVICs are the reference task of choice for normalizing EMG, they also needed to be normalized because they were also the task exercise to assess neuromuscular function recovery. In such a situation, where the maximal task is the also the exercise task, it is appropriate therefore to normalize the muscle activity (RMS EMG amplitude) of the maximal exercise task to a reliable (i.e., low within- and between-day variation) submaximal task (28). In the present study we employed a bilateral sit-to-stand task, where participants sat in a free-standing chair and, upon instruction, stood up from the seated position. Seat position was standardized among participants and between conditions, with the sit-to-stand task completed at a rate of 1 s to go from seated to standing upright via a metronome. The peak RMS EMG amplitude from this task (200 ms in total, 100 ms either side of the single instantaneous peak data point) was used to normalize the RMS EMG amplitude from each visit’s MVICs. EMG50 denotes EMG amplitude during the MVIC at 50° knee flexion, and EMG90 denotes EMG amplitude during the MVIC at 90° knee flexion.

### Creatine Kinase

Creatine kinase (CK) levels were assessed before any exercise including warm-up at baseline, after exercise (+5 min), and at 24 and 48 h after exercise. After cleansing with an alcohol swab, fingertip skin was pierced with a standard lancet and capillary blood was collected in a 32-μL heparinized Reflotron capillary tube (Selzer Labortechnik, Germany). The blood sample was then applied to the Reflotron creatine kinase strip (Roche, Germany) and immediately analyzed for creatine kinase concentration via a Reflotron Plus system (Roche, Germany).

### Muscle Soreness Rating

Participants were asked to rate their perception of vastus lateralis muscle soreness before any exercise including warm-up at baseline, immediately after exercise (+ 5 min), and at 24 and 48 h after exercise. Participants rated muscle soreness on a continuous visual analog scale scored from 0 to 100, with 0 being no soreness at all and 100 being the most unbearable soreness imaginable.

### Resistance Training Protocol

Participants performed four sets of eight repetitions of maximal voluntary isometric contractions at the evaluated joint angle. Contractions were 3 s in duration (audibly and visually timed and instructed by the dynamometer computer system) with a 2-s passive rest between reps. There was 2 min of passive rest between sets. During exercise, instantaneous peak torque of each contraction was recorded as detailed in *Knee Extensor Maximal Voluntary Isometric Contraction*, with EMG amplitude and spectral frequencies of each contraction analyzed as in *Electromyography*. Internal quadriceps muscle force was calculated by dividing the quadriceps maximal torque by the patella tendon moment arm (16).

### Statistics

JASP v 0.17.3 was used to perform all descriptive and inferential statistical analyses. First, data parametricity was established through checking the usual filters of data level, independence of samples, normal distribution (with the Shapiro–Wilk test), and equal variance (with Levene’s test). In addition, the presence of any outliers was checked with stem-and-leaf plots. To contrast resistance exercise parameters (i.e., peak torque, EMG amplitude and frequencies, internal muscle forces), paired *t* tests were used, except for architecture, which was assessed by a two-way ANOVA [main effect of muscle length and main effect of contraction status (relaxed and maximally contracted)]. Cohen’s *d* was used to denote effect size in paired-samples *t* tests, where 0.2 = small, 0.5 = medium, and 0.8 = large. For comparisons of interest, mean difference and 95% confidence interval (95% C.I.) are also presented. Key outcome data (muscle strength, EMG amplitude, creatine kinase, and self-perceived muscle soreness) were reduced into changes relative to baseline and analyzed by using a two-way repeated-measures ANOVA to determine the impact of exercise angle on the muscle recovery time course. Statistical significance for these key outcomes was accepted at an alpha ≤ 0.05, effect size for ANOVAs was taken as partial eta squared ($\eta_{p}^{2}$; 0.01 = small; 0.06 = medium; and 0.14 = large effect), and significant study power was accepted at β ≥ 0.8.

## RESULTS

### Acute Resistance Exercise Protocol Results

#### Electromyography and peak torque.

**Figure 2.**
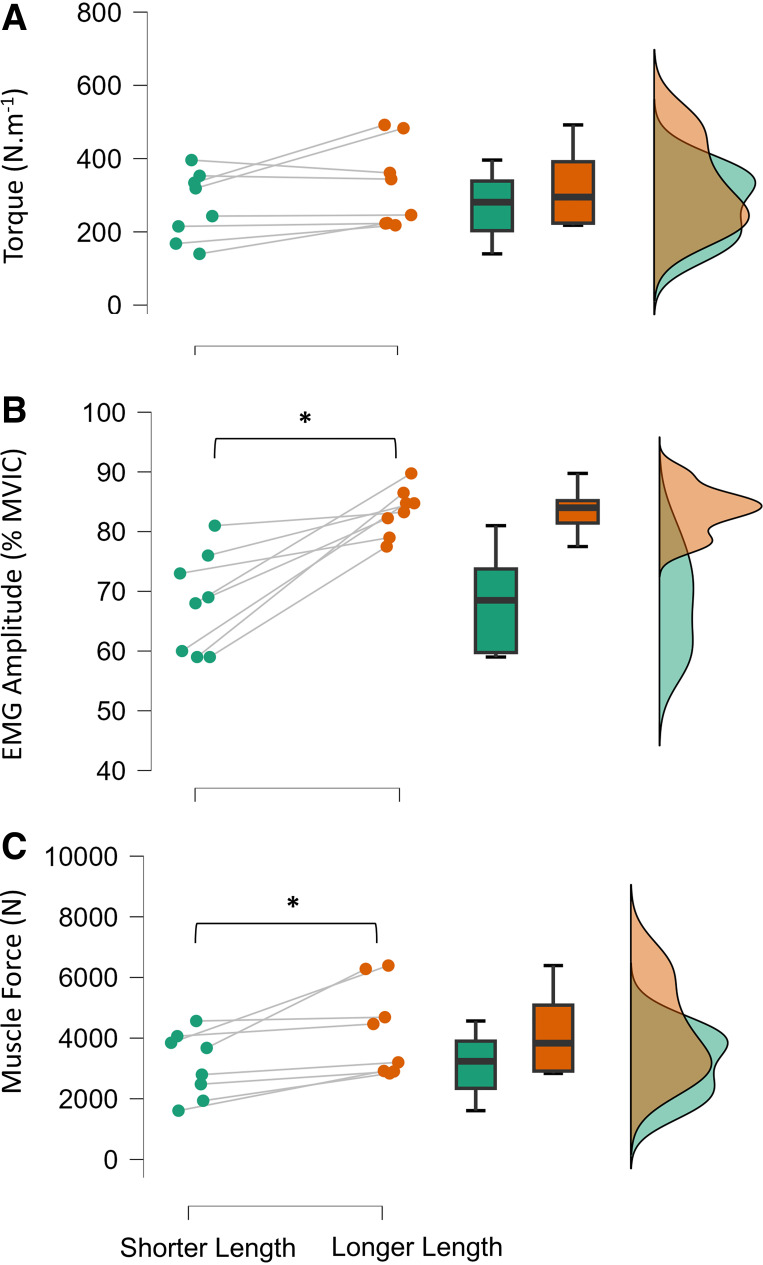
Neuromuscular impact of resistance exercise at shorter muscle length (SL) and longer muscle length (LL) on quadriceps torque (*A*), muscle activation (*B*), and internal quadriceps muscle forces (*C*). EMG, electromyogram; MVIC, maximal voluntary isometric contraction. Data are presented as a box-and-whisker plot [“box” = lower quartile (*bottom* line), median (horizontal line), and upper quartile (*top* line) values; “whiskers” = minimum (*bottom*) and maximum (*top*) data values]. *n* = 8. *Significant difference between SL and LL exercise conditions.

EMG amplitude (*P* = 0.002, *d* =1.66; mean difference −15%; 95% C.I. −23 to −7%), internal muscle force (*P* = 0.017, *d* = 1.10; mean difference −2,178 N; 95% C.I. −3,829 to −527 N), and peak EMG amplitude (*P* = 0.014, *d* = 1.15; mean difference −7%; 95% C.I. −13 to −2%) were greater in the LL versus SL condition. Peak torque (*P* = 0.08), mean frequency (*P* = 0.45), and median frequency (*P* = 0.39) did not differ between protocols (Table 2), thereby suggesting that the external training load was matched although the internal work by the muscle differed (see Fig. 2) as expected.

**Table 2.** Peak RMS EMG and spectral frequencies in shorter-muscle length and longer-muscle length conditions

	SL	LL	*P* Value
Peak EMG amplitude, % MVC	89 (86 to 93)	97 (92 to 102)*	0.014
Mean frequency, Hz	144 (125 to 162)	135 (118 to 159)	0.56
Median frequency, Hz	104 (94 to 109)	103 (95 to 107)	0.88

Data are mean [95% confidence interval (95% C.I.)]; *n* = 8. Note that the training parameters reported are the average values across 4 sets. EMG, electromyography; LL, longer muscle length; MVC, maximal voluntary contraction; RMS, root mean square; SL, shorter muscle length. *Significantly greater than SL.

#### Muscle architecture.

There was no main effect of contraction status (i.e., relaxed vs. MVIC) on muscle thickness (*P* = 0.43) or exercise angle (*P* = 0.32) or contraction status × exercise angle interaction (*P* = 0.89). There was a main effect of contraction status, with PA significantly greater (*P* < 0.001, $\eta_{p}^{2}$ = =0.91) in MVIC state. There was also a main effect of exercise angle, with PA in SL greater (*P* < 0.001, $\eta_{p}^{2}$ = 0.88; mean difference −2.1; 95% C.I. 1.4 to 2.7) than in LL, with no contraction status × exercise angle interaction (*P* = 0.08). There was a main effect of contraction status, with fascicle length significantly greater (*P* < 0.001, $\eta_{p}^{2}$ = 0.94) in MVIC state. There was also a main effect of exercise angle, with fascicle length in LL greater (*P* = 0.003, $\eta_{p}^{2}$ = 0.72; mean difference 1.1; 95% C.I. −1.8 to −0.5) than in SL, with no contraction status × exercise angle interaction (*P* = 0.055). Taken together, these observations show that, as expected, muscle architecture reflects the external angle of the knee both at rest and during maximum voluntary efforts (Table 3).

**Table 3.** Muscle architecture parameters in shorter-muscle length and longer-muscle length conditions in relaxed and maximally contracted states

Architecture Parameter	SL	LL	*P* Value
Muscle thickness, cm
Relaxed	2.20 (1.85 to 2.53)	2.12 (1.80 to 2.42)*§	<0.001, 0.023
MVIC	2.20 (1.88 to 2.52)	2.19 (1.83 to 2.54)§	0.008
Pennation angle, °
Relaxed	17.2 (16.2 to 18.0)	14.7 (14.0 to 15.2)§	0.005
MVIC	19.3 (18.2 to 20.3)	17.7 (16.4 to 18.8)*§	<0.001, 0.019
Fascicle length, cm
Relaxed	8.7 (7.9 to 9.5)	9.8 (8.5 to 11.0)§	0.008
MVIC	7.4 (6.6 to 8.0)	8.6 (7.5 to 9.7)*§	<0.001, 0.006

Data are mean [95% confidence interval (95% C.I.)]; *n* = 8. LL, longer muscle length; MVIC, maximal voluntary isometric contraction; SL, shorter muscle length. *Significant difference between relaxed and MVIC conditions within the same exercise angle condition; §significant difference between SL and LL.

### Recovery Time Course Comparison: Shorter- vs. Longer-Muscle Length Resistance Training

#### Peak torque.

**Figure 3.**
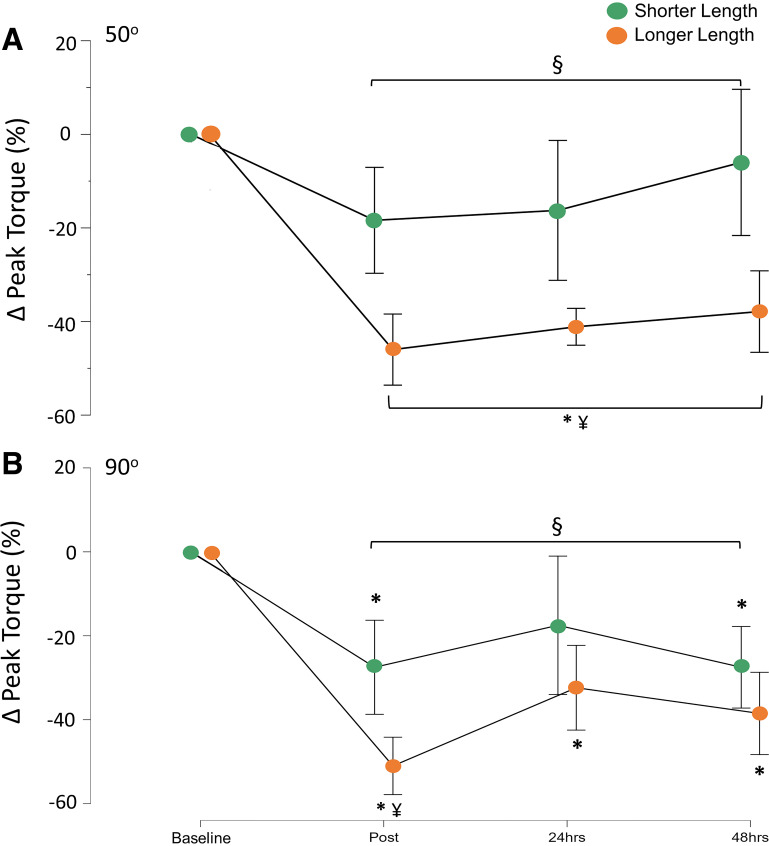
Time course of peak torque recovery in peak torque produced at 50° knee flexion (PT50; *A*) and peak torque produced at 90° knee flexion (PT50; *B*) immediately after (Post) and 24 h and 48 h after exercise at shorter muscle length (SL) and longer muscle length (LL). Data are mean [95% confidence interval (95% C.I.)]. *n* = 8. *Significant difference from baseline (main effect of time). §Significant difference (*P* < 0.05) between exercise conditions (main effect of exercise angle). ¥Significant difference (*P* < 0.001) between conditions (main effect of exercise angle × time interaction).

The time course of PT50 showed that there was a main effect of time (*P* < 0.001, $\eta_{\text{p}}^{2}$ = 0.58) and exercise angle (*P* = 0.002, $\eta_{p}^{2}$ = 0.75; mean difference 21%; 95% C.I. 10 to 32) and exercise angle × time interaction (*P* < 0.001, $\eta_{p}^{2}$ = 0.59; Fig. 3*A*). Post hoc contrasts for PT50 after exercise in SL show no main effect of time, with no differences compared to baseline immediately after exercise (Post) (*P* = 0.48), 24 h after exercise (+24 h) (*P* = 0.93), and 48 h after exercise (+48 h) (*P* = 0.99). However, post hoc contrasts for PT50 after exercise in LL showed that the peak torque was reduced at Post, +24 h, and +48 h (all *P* < 0.001). Post hoc contrasts for main effect of exercise angle × time interaction showed that the reduction in torque was greater in LL at each respective time point at Post (*P* = 0.004; mean difference 27%; 95% C.I. 5 to 49), +24 h (*P* = 0.011; mean difference 25%; 95% C.I. 46 to 6), and +48 h (*P* < 0.001; mean difference 32%; 95% C.I. −6 to 49).

PT90 revealed a main effect of time (*P* < 0.001, $\eta_{\text{p}}^{2}$ = 0.62), effect of exercise angle (*P* = 0.016, $\eta_{p}^{2}$ = 0.58; mean difference 12%; 95% C.I. 3 to 21%), and exercise angle × time interaction (*P* = 0.005, $\eta_{p}^{2}$ = 0.45; Fig. 3*B*). Post hoc contrasts for PT90 after exercise in SL showed a main effect of time compared with baseline at Post (*P* = 0.038) and +48 h (*P* = 0.027) but not at +24 h (*P* = 0.37). Post hoc contrasts for PT90 after exercise in LL showed that peak torque was reduced at Post (*P* < 0.001), +24 h (*P* = 0.011), and +48 h (*P* < 0.002). Post hoc contrasts for main effect of exercise angle × time interaction showed that the reduction in torque was greater in LL compared with SL at Post (*P* = 0.004; mean difference 23%; 95% C.I. 4 to 42%) but not at +24 h (*P* = 0.3) or +48 h (*P* = 0.99).

#### EMG.

**Figure 4.**
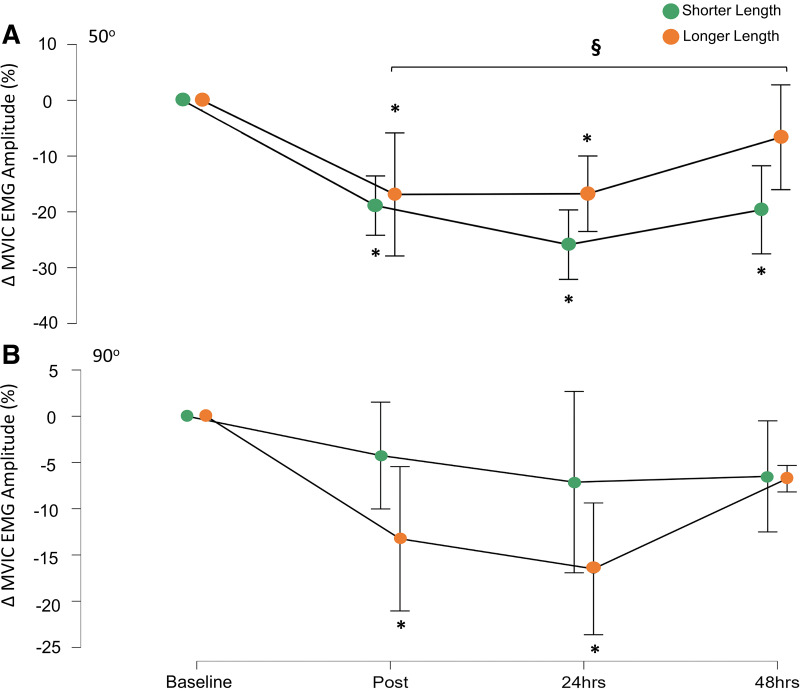
Time course of electromyographic (EMG) amplitude recovery in EMG amplitude at 50° knee flexion (EMG50; *A*) and EMG amplitude at 90° knee flexion (EMG90; *B*) immediately after (Post) and 24 h and 48 h after exercise at shorter muscle length (SL) and longer muscle length (LL). Data are mean [95% confidence interval (95% C.I.)]. *n* = 8. *Significant difference (*P* < 0.05) from baseline. §Significant difference (*P* < 0.05) between exercise conditions (main effect of exercise angle).

The time course of EMG50 amplitude showed that there was a main effect of time (*P* < 0.001, $\eta_{p}^{2}$ = 0.71) and exercise angle (*P* = 0.002, $\eta_{p}^{2}$ = 0.77; mean difference −6%; 95% C.I. −9 to −3%) but not exercise angle × time interaction (*P* = 0.20; Fig. 4*A*). Post hoc contrasts for EMG50 after exercise in SL showed a main effect of time compared with baseline at Post (*P* = 0.005), +24 h (*P* < 0.001), and +48 h (*P* = 0.002). Post hoc contrasts for PT90 after exercise in LL showed that peak torque was reduced at Post (*P* = 0.018) and +24 h (*P* = 0.019) but not at +48 h (*P* = 0.99). The main effect of exercise angle showed that EMG50 was reduced more in SL compared with LL.

The time course of EMG90 amplitude showed that there was a main effect of time (*P* = 0.002, $\eta_{p}^{2}$ = 0.50) but not exercise angle (*P* = 0.50) or exercise angle × time interaction (*P* = 0.07; Fig. 4*B*). Post hoc contrasts for EMG90 after exercise in SL showed no main effect of time compared with baseline at Post, +24 h, and +48 h (all *P* = 0.99). Post hoc contrasts for EMG90 after exercise in LL showed that peak torque was reduced at Post (*P* < 0.013) and +24 h (*P* < 0.001) but not at +48 h (*P* = 0.99).

#### Creatine kinase.

For SL or LL there was no main effect of time (*P* = 0.44) on creatine kinase level changes; thus no post hoc contrasts were attempted. It is nonetheless noteworthy that Table 4 reveals that in absolute terms in LL there was a difference in CK levels between baseline and the three follow-up points (Post, +24 h, +48 h) yet there were no differences in absolute CKs at any time point in SL (Table 4).

**Table 4.** Creatine kinase levels and muscle soreness rating time course after exercise in shorter-muscle length and longer-muscle length conditions

Recovery Indexes	Time Point	SL	LL	*P* Value
CK	Baseline, U/L	164 (106 to 221)	136 (68 to 88)	
	Post, U/L	171 (121 to 219)	159 (102 to 214)*	0.027
	Post-24 h, U/L	265 (152 to 335)	258 (108 to 408)*	0.008
	Post-48 h, U/L	244 (132 to 152)	209 (121 to 295)*	0.027
	Δ(Pre–Post), %	9 (−3 to 21)	17 (−10 to 219)	
	Δ(Pre–Post-24 h), %	73 (14 to 132)	103 (−4 to 188)	
	Δ(Pre–Post-48 h) %	73 (−23 to 169)	57 ± 89	
Soreness	Baseline, mm	23 (14 to 31)	23 (12 to 34)	
	Post, mm	45 (32 to 57) *	43 (29 to 55)*	<0.001
	Post-24 h, mm	31 (20 to 40)	33 (22 to 43)	
	Post-48 h, mm	22 (16 to 27)	24 (15 to 32)	
	Δ(Pre–Post), %	147 (−3 to 217)*	120 (−10 to 175)*	0.008
	Δ(Pre–Post-24 h), %	52 (−14 to 132)*	75 (−13 to 219)*	0.017
	Δ(Pre–Post-48 h), %	22 (−23 to 169)**	23 (−4 to 118)**§	0.032, 0.004

Data are mean [95% confidence interval (95% C.I.)]; *n* = 8. CK, creatine kinase; LL, longer muscle length; Post, immediately after exercise; Post-24 h, 24 h after exercise; Post-48 h, 48 h after exercise; Pre, baseline; SL, shorter muscle length. *Significantly different from baseline; §significantly different from ΔPre–Post; **significantly different from ΔPre–Post-24 h.

#### Perception of muscle soreness.

The time course of muscle soreness showed that there was a main effect of time (*P* < 0.001, $\eta_{p}^{2}$ = 0.47) but not exercise angle (*P* = 0.56) or exercise angle × time interaction (*P* = 0.62; Table 4). Post hoc contrasts revealed that the pooled relative changes at Post (136%) and +24 h (63%) were greater (*P* = 0.042) than that at baseline but not at +48 h (25%). Furthermore, soreness at Post was greater (*P* = 0.033) than +24 h and +48 h, and +24 h was greater than +48 h (*P* = 0.034; Table 3).

## DISCUSSION

The primary aim of this study was to investigate whether there is a difference in the time course of recovery of neuromuscular characteristics, muscle damage, and soreness depending on whether prior isometric resistance exercise was carried out at a shorter or longer muscle length. We demonstrate, for the first time in the quadriceps, that there is joint angle specificity in the recovery of neuromuscular characteristics after isometric resistance exercise at different muscle lengths. Isometric resistance exercise at longer muscle lengths resulted in greater impairment of neuromuscular function at both shorter and longer muscle lengths during recovery, compared with exercising at shorter muscle lengths. Furthermore, our acute results suggest that these are likely due to larger internal forces, muscle activation, and fascicle stretch during maximal isometric exercise at longer muscle lengths. The study also found that the impairment of neuromuscular function was not reflected in creatine kinase or muscle soreness variables.

Manipulation of joint angle during resistance exercise training has previously been shown to have profound acute physiological, mechanical, and performance-related consequences for the exercising muscle, including increased voluntary activation (6–8), muscle activity (9), muscle oxygen consumption (6, 10), and development of fatigue (11–15) at longer muscle lengths. The results from the present study reaffirm these findings of higher physiological and mechanical demands at longer muscle lengths, as our acute data during the resistance training demonstrated significantly higher muscle activation during torque production at longer muscle lengths. This was accompanied by significantly higher internal forces generated by the quadriceps at longer muscle lengths despite no differences in the external torque production due to the patella tendon moment arm length at each joint angle. In addition, our muscle architectural measurements demonstrated that both at rest and at MVIC muscle fascicle lengths were significantly longer at the longer-muscle length versus shorter-muscle length joint angles. These data clearly demonstrate that isometric exercise performed at longer muscle lengths in the quadriceps has increased neuromechanical demands in the form of internal muscle force, muscle activity, and fascicle stretch.

With increased demands on the muscle during maximal isometric longer-muscle length exercise compared to shorter muscle lengths, the magnitude of impairment during the time course of muscle function recovery at shorter muscle lengths was greater after exercise at longer muscle lengths in the present study. Previous reports from Philippou and colleagues (22, 23) in seven healthy young men found that performing maximal isometric contractions at a longer muscle length (155° elbow angle) resulted in a sustained decrement of maximal isometric force at shorter muscle lengths (50–90° elbow angle) for up to 4 days after exercise, whereas maximal isometric force at longer muscle lengths was either unaffected or had recovered to baseline levels within the 4-day recovery period. The authors also reported that resting elbow joint angle and the joint angle of maximal force production shifted to longer muscle lengths during this time frame. These findings agree somewhat with the present study; however, in the present study performed in the quadriceps we have reported that there was also a significant decrement in MVC at both longer and shorter muscle lengths after resistance exercise at longer muscle lengths. The aforementioned studies of Philippou et al. (22, 23), however, did not have accompanying data on the time course of recovery of torque-angle relationships after maximal isometric exercise at shorter muscle lengths in the elbow flexors. Allen et al. (24) performed a series of experiments investigating the relationships between maximal isometric resistance exercise at shorter (90° elbow angle) and longer (155° elbow angle) muscle lengths, again in the elbow flexors. A combination of 10 healthy young males and females (exact breakdown of sex not provided in the study) performed a single set of thirty 3.5-s-long maximal isometric contractions, separated by 1-min recovery between contractions, at shorter and longer muscle lengths. Their results showed that exercise in both muscle length conditions resulted in significant reductions in maximal force immediately after and at 2 h, 4 h, and 24 h after exercise at their respective joint angles. The authors noted a significant effect of exercise condition at each of the recovery time points, with the change in maximal force relative to baseline significantly higher in the longer-muscle length condition compared with shorter muscle length. It is important to note that these comparisons of MVICs measured during the recovery time frame were matched to the preceding exercise condition only and then compared (i.e., after exercise at 90° MVICs were only assessed at 90°, and after exercise at 155° MVICs were assessed at 155°, with the effects on function at different muscle lengths not investigated). Therefore, the study of Allen et al. (24) does not allow the identification of the full impact of exercising at different muscle lengths on resulting recovery of the torque-angle relationships. This highlights the uniqueness of the present study design in comparison to the previous studies of Philippou et al. (22, 23) and Allen et al. (24), where we employed two exercising conditions at both shorter and longer muscle lengths as well as assessing the time course of muscle function at both shorter and longer muscle lengths. This was also important because the results of the present study found that maximal isometric training at shorter muscle lengths did not impair function at all at shorter muscle lengths. This is in contrast to the study of Allen et al. (24), who found that muscle function was impaired at shorter muscle lengths immediately after and 2 h, 4 h, and 24 h after exercising at short muscle lengths. One of the reasons may have been the differences in the protocols employed between the studies. As mentioned above, Allen et al. (24) conducted a set of thirty 3.5-s-long maximal isometric contractions, approximating a total of 105 s of work over ∼30 min. In the present study, we employed four sets of eight maximal 3-s-long contractions, separated by 2-s recovery between reps and 2-min recovery between sets. Our participants therefore completed 96 s of work in ∼10 min. The 1-min rest between MVCs in the study of Allen et al.(24) may have provided the participants an opportunity to largely maintain neuromuscular recovery of torque between contractions [see Fig. 2A of Allen et al. (24)], whereas the more contemporary training prescription in the present study did not afford this. The maintenance of higher torque in Allen et al. (24), despite an exercise volume similar to the present study, may have resulted ultimately in higher muscle damage and loss of function compared with the present study.

A second important aspect of this study was that all these previous studies were performed in the elbow flexors, whereas the present study provides information for the quadriceps muscle group. The transfer of evidence from elbow flexors to quadriceps is limited, as the groups have different torque-angle relationships over different active ranges. For example, in the elbow flexors an elbow angle of 90° is used to represent shorter muscle lengths on the ascending limb, or muscle lengths close to the optimal angle of force production in and around the plateau of the torque-angle relationship, and an elbow angle of 155° is used to represent function at longer muscle lengths on the descending limb of the torque-angle relationship. This is in contrast to the quadriceps, where 90° knee flexion represents longer muscle lengths on the descending limb of the torque-angle relationship and 50° represents shorter muscle lengths on the ascending limb of the torque-angle relationship. Based on the above results, future studies may want to investigate how dynamic tasks, at various velocities, are performed at shorter muscle lengths after resistance exercise at either shorter or longer muscle lengths.

Both muscle function and muscle activity were significantly reduced at shorter muscle lengths at each postexercise time point following exercise at longer muscle lengths. Muscle function at longer muscle lengths was also impacted to a greater degree by exercise at longer versus shorter muscle lengths, yet the EMG results did not show any exercise condition effects. This suggests that whereas there may in fact be a specific neuro-modulatory impact on muscle function following maximal isometric resistance exercise at short muscle lengths (1), neural factors relating to muscle activity do not explain the joint angle-specific decrements in function following maximal isometric resistance exercise at longer muscle lengths. However, Jones et al. (20) showed that low-frequency fatigue was greater in the quadriceps after isometric exercise at longer versus shorter muscle lengths. Therefore, there may be a task-specific impact on neuromuscular function after isometric exercise at different muscle lengths (i.e., fatigue related to repeated muscle contractions compared with maximal force output contractions).

Increased impairment of muscle function at shorter muscle lengths following longer-muscle length exercise compared with shorter-muscle length exercise may be explained by sarcomere mechanical factors. Philippou et al. (22, 23) and Jones et al. (20) suggest that resistance exercise at longer muscle lengths results in “overstretched” sarcomeres located toward the middle of muscle fibers, i.e., at the muscle belly, where they are subsequently forced to work on the descending limb of the sarcomere length-tension relationship, producing less force. This nonuniform distribution of sarcomere lengths and damage to sarcomeres following elongation is outlined in the “popping sarcomere” hypothesis (25). This may help explain the why muscle force was reduced at shorter muscle lengths after exercise at longer muscle lengths in the present study but force was not reduced at shorter muscle lengths after exercise at shorter muscle lengths. The observation from the present study that isometric exercise at shorter muscle lengths resulted in a smaller magnitude of functional loss at longer muscle lengths than isometric exercise performed at longer muscle lengths is, to the authors’ knowledge, the first report of this in the literature. This may possibly be more easily explained by factors such as higher internal forces, higher muscle activations, and fascicle stretch that are reported in the present study and have been shown to impact recovery in previous studies (17, 18).

Resistance exercise at longer muscle lengths incurs greater oxygen consumption, muscle activation, and opening of stretch-activated ion channels and therefore has the potential to increase the magnitude of muscle damage after exercise. Previous studies such as that of Philippou et al. (22) have shown that indirect markers of muscle damage (CK) are significantly increased 24 h after training at longer muscle lengths in the elbow flexors but return to baseline by 48 h. As previously highlighted, Philippou et al. (22) did not have a comparative exercise condition at shorter muscle lengths. In the present study, absolute CK levels were significantly increased immediately after and at 24 h and 48 h after exercise following training at longer muscle lengths. However, despite the within-condition significant changes at longer muscle lengths, there were no between-condition differences in CK levels in absolute or relative terms. Therefore, one should exercise caution in the interpretation of the present results, as they do not conclusively suggest that muscle damage is any greater at longer compared with shorter muscle lengths. Between-group differences would be difficult to decipher in the data because of variability in absolute CK values, as evidenced by the elevated standard deviations of the results in the present study.

Muscle soreness was also significantly increased immediately after exercise in both exercise conditions but surprisingly was not different from baseline at 24 h or 48 h after exercise. This contrasts with Philippou et al. (22), who showed that muscle soreness had not returned to baseline after 4 days of recovery after resistance exercise (RE) at longer muscle lengths in the elbow flexors. However, in the study of Philippou et al. (22), the authors employed a much more severe exercise prescription compared with the present study, where participants completed two sets of 25 maximal isometric contractions, 10 s in duration, with 20 s of rest between contractions and 5 min rest between sets. This accumulates to 500 s of maximal force production, which is approximately five times higher than the 96 s of maximal force production carried out in the present study.

Maximal isometric resistance exercise impairs neuromuscular function for several days after exercise at longer and shorter muscle lengths. However, if the preceding exercise is performed at longer muscle lengths, neuromuscular function at shorter muscle lengths is impaired during recovery to a greater degree compared with preceding exercise at shorter muscle lengths. Despite this, muscle damage and perception of muscle soreness appear to be largely unaffected by length-specific resistance exercise. Although it is important to acknowledge the results of the present study to inform decision making around the acute effects of training at longer and shorter muscle lengths, one must not forget that performing sustained resistance training, whether it be isometric or dynamic at longer muscle lengths, leads to superior morphological, architectural, functional, and tendon mechanical properties at longer muscle lengths versus shorter muscle lengths (4, 5, 29–31).

### Study Strengths and Limitations

The present study has several important strengths worth noting. The study used a within-subjects design, a comparison of two exercise angles commonly used at either end of habitual training range of motion (ROM) and how training at each angle impacts muscle structure and function at the each of the joint angles, providing a strong overview of these parameters. Furthermore, the study analyzed a good range of objective and subjective markers for assessing DOMS (functional, biochemical, and self-reports). Finally, the study was powered to detect the primary outcome and showed some moderate and large effect sizes. There are nonetheless two key limitations within the present study. First, the a priori sample size was calculated from previous studies using the primary outcome measure of maximal force. Although the sample size was sufficient in regard to this variable, the study may not have necessarily been adequately powered to detect differences in other variables. For instance, creatine kinase is highly variable between individuals (32), as reflected in the present large standard deviations in our results. The second limitation is associated with the EMG normalization approach. Indeed, although a maximal isometric voluntary contraction is an established normalization procedure for EMG, the MVIC was also the functional test task during the recovery period, rendering it suboptimal as an EMG normalization task (28). Electrical stimulation and normalization to the M wave is also a common approach; however, this technology was not available to the authors during data collection. In the present study, EMG amplitude normalization was conducted with submaximal, dynamic contractions. Besomi et al. (33) caution against this because the normalization task may be more difficult to replicate/standardize each day. It is, however, noteworthy that we took the utmost care in the approach to the EMG normalization for these reasons, choosing a task (sit-to-stand) that has excellent reliability, is very familiar to the participants, is relatively low in intensity (i.e., therefore unlikely to be affected by soreness from exercise), and was controlled via a metronome.

### Conclusions

Maximal isometric resistance exercise impairs neuromuscular function at shorter and longer muscle lengths to a greater magnitude if the preceding exercise is performed at longer muscle lengths compared with preceding exercise at shorter muscle lengths. Muscle function appears to be reduced only at longer muscle lengths when preceding exercise is carried out at shorter muscle lengths. Practitioners and clinicians may use the results of the present study to inform choice of pre-/rehabilitation exercise prescription. For example, if an isometric training bout is to be performed within 24–48 h of a preceding training session at different joint angles, practitioners should be aware that there may be a specific and expected decreased performance capacity at that particular joint angle if monitoring force output.

## DATA AVAILABILITY

Data are available upon request.

## SUPPLEMENTAL MATERIAL

10.6084/m9.figshare.25270375 Supplemental Fig. S1: https://doi.org/10.6084/m9.figshare.25270375.

## DISCLOSURES

No conflicts of interest, financial or otherwise, are declared by the authors.

## AUTHOR CONTRIBUTIONS

G.M. conceived and designed research; G.M. performed experiments; G.M. and G.O.-P. analyzed data; G.M. interpreted results of experiments; G.M. prepared figures; G.M. drafted manuscript; G.M. and G.O.-P. edited and revised manuscript; G.M. and G.O.-P. approved final version of manuscript.
